# Web-Based Learning for General Practitioners and Practice Nurses Regarding Behavior Change: Qualitative Descriptive Study

**DOI:** 10.2196/45587

**Published:** 2023-07-27

**Authors:** Lauren Raumer-Monteith, Madonna Kennedy, Lauren Ball

**Affiliations:** 1 Nutrition and Dietetics School of Health Sciences and Social Work Griffith University Gold Coast Australia; 2 Prevention Strategy Branch Queensland Health Brisbane Australia; 3 Centre for Community Health and Wellbeing University of Queensland Brisbane Australia

**Keywords:** continuing professional development, continuing medical education, web-based, e-learning, behavior change, general practitioner, practice nurse, nurse, medical education, professional development, general practice, web-based learning, remote learning, adoption, perspective, health care professional

## Abstract

**Background:**

Supporting patients to live well by optimizing behavior is a core tenet of primary health care. General practitioners and practice nurses experience barriers in providing behavior change interventions to patients for lifestyle behaviors, including low self-efficacy in their ability to enact change. Web-based learning technologies are readily available for general practitioners and practice nurses; however, opportunities to upskill in behavior change are still limited. Understanding what influences general practitioners’ and practice nurses’ adoption of web-based learning is crucial to enhancing the quality and impact of behavior change interventions in primary health care.

**Objective:**

This study aimed to explore general practitioners’ and practice nurses’ perceptions regarding web-based learning to support patients with behavior change.

**Methods:**

A qualitative, cross-sectional design was used involving web-based, semistructured interviews with general practitioners and practice nurses in Queensland, Australia. The interviews were recorded and transcribed using the built-in Microsoft Teams transcription software. Inductive coding was used to generate codes from the interview data for thematic analysis.

**Results:**

In total, there were 11 participants in this study, including general practitioners (n=4) and practice nurses (n=7). Three themes emerged from the data analysis: (1) reflecting on the provider of the Healthy Lifestyles suite; (2) valuing the web-based learning content and presentation; and (3) experiencing barriers and facilitators to using the Healthy Lifestyles suite.

**Conclusions:**

Provider reputation, awareness of availability, resources, content quality, usability, cost, and time influence adoption of web-based learning. Perceived quality is associated with culturally tailored information, resources, a balance of information and interactivity, plain language, user-friendly navigation, appealing visual presentation, communication examples, and simple models. Free web-based learning that features progress saving and module lengths of less than 2 hours alleviate perceived time and cost barriers. Learning providers may benefit by including these features in their future behavior change web-based learning for general practitioners and practice nurses.

## Introduction

Web-based technologies are a rapidly growing tool for learning in continuing professional development. Continued learning improves the knowledge, skills, and performance of general practitioners (GPs) and practice nurses (PNs), enhancing clinical competency and quality of care [1]. Opportunities for continued learning have expanded considerably in recent years, with a 42% increase in clinicians (including GPs) opting for web-based offerings in 2018 (prepandemic) [2]. Web-based learning is regarded as effective as traditional modalities for improving professional competence and skill development [3,4]. Unfortunately, GPs and PNs experience many barriers to web-based learning [5-11]. Given the rise of more technologically inclined clinicians and the effects of the pandemic, web-based modalities can be predicted to increase and should be given further consideration by learning providers.

Behavior change is a complex practice area in primary health care and comprises any activities used by health professionals to elicit changes in patient behaviors [12]. While GPs and PNs are encouraged through practice guidance to advocate for healthy lifestyles [13], only 8% of all interactions involve behavior change [14]. Behavior change interventions in Australian primary health care are insufficient in frequency [14], as GPs and PNs experience many barriers to providing behavior change interventions to their patients, including inadequate skills and knowledge [15-18], lack of time [15-19], and insufficient resources [5,15,18,20]. While learning programs for behavior change have been shown to reduce these barriers and improve behavior change skills [21,22], GPs and PNs lack the opportunities to upskill in areas such as behavior change [15]. Thus, GPs and PNs need support to develop the skills required to facilitate behavior change in partnership with their patients, which may assist in addressing the trajectory of chronic disease.

Despite the rise of web-based offerings, no studies have exclusively explored the views of GPs and PNs regarding web-based learning to support patients with behavior change. Particularly with the increasing prevalence of chronic disease [23], it is vital to understand the factors influencing Australian GPs’ and PNs’ selection of web-based learning to ensure the delivery of impactful behavior change interventions. Queensland Health recognizes the importance of primary health care and has developed a web-based Healthy Lifestyles suite, hosted by Insight, to facilitate learning and assist health professionals in motivating patients to make healthier choices [24]. However, the acceptability and feasibility of the Healthy Lifestyles suite has yet to be established. The aim of this study was to explore GPs’ and PNs’ perceptions regarding web-based learning to support patients in improving their lifestyle behaviors through behavior change. This information is essential to inform future web-based learning design and improve the adoption and implementation of behavior change interventions in primary health care.

## Methods

### Overview and Aim

A descriptive, cross-sectional design was used involving semistructured interviews, as contextualized in qualitative research [25]. This study aimed to explore GPs’ and PNs’ perceptions regarding web-based learning to support patients with behavior change. The study comprised two objectives: (1) to explore GPs’ and PNs’ perceived needs for web-based learning for behavior change related to lifestyle behaviors; and (2) to explore GPs’ and PNs’ perceptions of the acceptability and feasibility of the Healthy Lifestyles suite.

The Healthy Lifestyles suite is a web-based learning toolkit for providing brief behavior change interventions and includes an introductory module and 8 topic modules covering nutrition and physical activity, smoking, healthy pregnancy, oral health and alcohol and other drugs [24].

### Interview Protocol Development and Pilot Testing

The interview protocol (Multimedia Appendix 1) was developed based on research conducted in the literature review and questions put forward by the research team [26]. The questions were designed to elicit responses pertaining to the study’s aims and objectives. All questions were reviewed by the research team. Three pilot interviews were conducted to determine the validity and effectiveness of the interview questions [26].

### Recruitment

Eligible participants were GPs and PNs employed within Queensland primary health care settings. For context, approximately 7500 GPs and approximately 3000 PNs were registered in Queensland during 2022 [27-29]. Purposive and snowball sampling methods were used to recruit prospective participants. The study was advertised through (1) a practice-based research network, (2) GP and PN social media groups, (3) primary health networks (PHN) newsletters, and (4) the Royal Australian College of General Practitioners (RACGP) research notice board. Participants were asked to contact the lead researcher via email with a preferred time and day for interviews. The lead researcher responded to the participants with all required information, hyperlinks to the Microsoft Teams website and the Healthy Lifestyles suite [24,30], and confirmation of interview date and time. The lead researcher scheduled the interviews and sent invitations to the participants on Microsoft Teams [30].

### Data Collection

Data were collected through semistructured interviews via Microsoft Teams [26,30]. Participants were asked to respond to questions from the interview protocol and use the “think aloud” method while completing the Brief Interventions: Nutrition and Physical Activity web-based module of the Healthy Lifestyles suite [31]. The think-aloud method was used to capture perceptions outside of questions included in the interview protocol [31]. The interviews were audio and video recorded.

All interviews were transcribed using the Microsoft Teams built-in transcription software [30,32]. The transcripts were then compared against the recorded interviews, with any identified errors corrected. The lead researcher emailed the transcript to each participant for verification of responses.

### Data Analysis

The verified transcripts were analyzed according to the Braun and Clark [33] 6-step framework for thematic analysis: (1) familiarity with the data, (2) generating initial codes, (3) searching for themes, (4) reviewing themes, (5) defining and naming themes, and (6) producing the report. An inductive approach was used, whereby themes were generated from the interview data [33]. All data analysis was completed by hand using Microsoft Excel. Data saturation was achieved after 9 interviews when no new themes could be derived from the transcriptional data [34]. Two additional interviews were performed to ensure thorough exploration and data quality [35].

### Triangulation

Researcher triangulation was used to verify the generated themes and subthemes [36]. Correspondence between the lead researcher and the research team continued until consensus was achieved upon themes and subthemes.

### Ethics Approval

Study approval was granted by the Griffith University Human Research Ethics Committee (2022/120) before the commencement of data collection and was conducted in accordance with the Australian National Statement on Ethical Conduct in Human Research. Prospective participants were offered AU $100 (approximately US $70) gift cards as reimbursement for their time. All participants were provided with an overview of the interview process, information sheet, and consent form via email correspondence prior to interview scheduling. Additionally, at the beginning of each interview, a verbal overview of the study was provided, and participant consent for the interview and analysis of recorded materials was obtained. Each recorded transcript was deidentified by assigning an identification number to ensure participant confidentiality. All interview recordings were deleted once transcript accuracy was established.

## Results

### Overview of Participants

In total, 11 participants completed a semistructured interview. The demographic characteristics of participants are described in Table 1. The sample predominantly comprised women (n=10). Of the 11 participants, 7 were employed as PNs and 4 as GPs. Their practice experience ranged from 2 to 35 years, with a mean of 15 years.

**Table 1:** Demographic characteristics of participants (n=11).

Characteristic			Participants, n
**Gender**			
	Male	1	
	Female	10	
	Other	0	
**Occupation**			
	Practice nurse	7	
	General practitioner	4	
**Years in practice**			
	<5	2	
	5-9	3	
	10-14	3	
	>30	3	

### Overall Themes

#### Overview of Themes

Three themes were identified from the data: (1) reflecting on the provider of the Healthy Lifestyles suite; (2) valuing the web-based learning content and presentation; and (3) experiencing barriers and facilitators to using the Healthy Lifestyles suite.

#### Theme 1: Reflecting on the Provider of the Healthy Lifestyles Suite

Many participants perceived Queensland Health as a reliable source of accurate information. Some participants appeared to trust the web-based module, given that it was created by a government institution.

I have a baseline sense of trust, given that this is an official module from Queensland Health. You know the information is correct.

P05, GP

Notably, 1 highly experienced GP voiced a contrasting view regarding Queensland Health’s reputation and the trust placed in the learning material.

Anything to do with Queensland Health sort of turns me off. I have...[a] little bit of a lack of trust with anything to do with Queensland Health.... I did wonder about what the motivation was for Queensland Health to have these modules.

P10, GP

A few PNs assumed there was an access barrier to web-based learning opportunities provided by Queensland Health. Although the Healthy Lifestyles suite is publicly available, some participants believed that they needed to be a staff member to access the learning materials, which prevented them from attempting to access learning opportunities through Queensland Health. As GPs and PNs must complete learning each year, this steered participants to pursue learning opportunities from other sources.

No, not unless you’re a member of Queensland Health...once you’re sort of locked out of that Queensland Health system...you sort of have to hunt and gather for your own CPD over multiple different, smaller websites.

P07, PN

Some participants expressed being aware of or already having an account with the Insight web-based learning platform. However, no GPs or PNs in the study seemed to be aware that the Healthy Lifestyles suite was available, despite being readily accessible.

I know I already had an account.... No, I was not [aware of the Healthy Lifestyles suite].

P02, PN

GPs and PNs suggested strategies they thought would improve awareness of the Healthy Lifestyles suite while thinking aloud. Many participants recommended advertising through the PHNs, the Australian Health Practitioner Regulation Agency, the RACGP, and other professional organizations, which appeared well known for promoting learning opportunities. Other suggestions included advertising through social media and primary health care practices.

Whether it’s [advertising] through the PHNs, they would be great in terms of putting these [web-based modules] up. The RACGP, having it [the Healthy Lifestyles suite] on their platform as well because I think a lot of people do their CPD through those sort of things as well.

P06, GP

I do get a lot of emails weekly from the PHNs and they’ll often have newsletters and updates.... A bulk email to practices.... Or perhaps something on Facebook?

P07, PN

#### Theme 2: Valuing the Web-Based Learning Content and Presentation

Participants highly valued information regarding Aboriginal and Torres Strait Islander populations. While thinking aloud, GPs and PNs drew upon their moral perspectives, as emphasis was placed on the importance of understanding diverse cultures and priority populations, which is relevant to culturally safe practice.

I didn’t know though, there was an Aboriginal Torres Strait Islander version of that [Australian Guide to Healthy Eating], which I really liked, so that was some new information for me.

P06, GP

I do find this thing about cultural identity interesting like, I think that is a gap missing sometimes.

P09, PN

The participants seemed to appreciate the resources such as pamphlets, brochures, and hyperlinks to reputable websites available within the web-based module, particularly for patient education purposes. Participants expressed that resources are generally challenging to find but are considered a powerful adjunct to consultations by facilitating conversations with patients.

It’s [the web-based module] got lots of resources that I can use to talk with my clients about for sure. Not just clients, but family and friends as well.

P02, PN

The amount of resources [is good value].... I find with a lot of things, when you’re informing patients, it’s hard to find resources.

P04, PN

One GP expressed while thinking aloud, the importance of Queensland-based resources. State-based resources were thought to increase GPs’ awareness of local programs available, which would be more relatable to their patients.

One of the best things about this whole resource is it’s all Queensland relatable. So you know that these things are within the state.

P06, GP

When asked about the tone of language within the web-based module, words such as *easy*, *basic* and *everyday English* were used. Participants seemed to believe that plain language was more engaging and applicable to many different learning styles. Plain language was described as more transferable across professions, particularly given the broad range of health professions requiring learning.

You’re better off using plain language that people can relate to. Keeps people’s attention more, but it’s still got the really healthy balance of keeping you on the edge of kind of always learning a bit more.

P01, PN

[The information is] easy to read...it’s quite broad in that it can reach many health care individuals from various backgrounds so.... I think it appeals to many different learning styles.

P08, PN

One participant expanded on language further, recalling a previous web-based learning experience containing many acronyms.

I did one [web-based learning] that had a key for all their acronyms in a separate appendix right down the bottom, but all throughout they were using all sorts of different acronyms. It just didn’t read well and it was confusing.

P07, PN

Web-based learning that consisted of predominantly text, interactive activities, or long videos was described as uninteresting and appeared to reduce GPs’ and PNs’ engagement. In contrast, a balance of information and interactivity seemed to increase engagement with the web-based module and facilitate greater learning. Many participants voiced their opinions that the web-based module contained an acceptable combination of information and interactivity that was conducive to learning, which is crucial given the nature of health professions.

I just like how it breaks it [the web-based module] up.... I don’t mind reading lots of text, but I found it just keeps my interest going for a bit longer and for me it’s almost like a little bit of a break. Something different to sort of switch my brain to.

P06, GP

It’s [the web-based module] not too thick and fast, and it’s not just a boring, barren, stream of words without anything in there to break them up. I think it’s a pretty good mix.

P07, PN

Responses implied that information accuracy is crucial for learning, which is pertinent to evidence-based practice. Many GPs and PNs perceived the use of statistics and references as an indicator of information accuracy. Additionally, some participants appeared to use their prior knowledge or experiences to determine the accuracy of the information.

I’ve just finished a public health masters, so all this [information] is pretty fresh, but it’s always nice to be reminded of it all.

P02, PN

Well, I guess it’s [the information] based on some sort of statistics and research. I believe it should be accurate.

P05, GP

I feel as if the content is very in tune with what sort of conversations actually happen behind closed doors in a treatment room.

P08, PN

When queried about the quality of the web-based module, participants referred to the use of pictures, colors, fonts, headers, and spacing. Referencing throughout the web-based module also seemed to increase the participants’ perception of quality.

I think its [the web-based module] of really good quality. A lot of good links...the color and presentation and information provided.

P04, PN

It’s [the web-based module] got nice big font [emphasis on big font], lots of headers, good spacing between each line. Lots of pictures.

P07, PN

Spacing and font size in the web-based module was perceived to increase usability, particularly for those with visual impairments or who spend substantial time on computers.

It’s [the web-based module] pretty well spaced out. Good for people with any visual impairments or difficulty with glasses or spending a lot of time on the computer.

P01, PN

Prior experience with technology appeared to increase the ability to navigate the web-based module with ease, particularly with the burgeoning use of telehealth since the start of the COVID-19 pandemic. Participants noted that including a side menu, scrolling, and clicking were easy to use, increasing the web-based module’s usability. Some participants stated that having a similar format to the Queensland Health COVID-19 web-based module increased familiarity and efficiency in navigating the Healthy Lifestyles web-based module.

All the nurses are familiar with the COVID vaccination training module that we’ve all had to do to give out COVID vaccines. It’s [the web-based module] very much the same format.

P03, PN

To me it [the web-based module] feels very intuitive to know, click, read, click, read. Like it does feel intuitive to scroll down, and then there’s the continue button. Alright, I’ve reached the end of that bit. Move on to the next bit.

P09, PN

PNs seemed to highly value the learning within the web-based module. Both PNs and some GPs believed that the learning would assist them in performing better patient assessments and reminded them of the importance of discussions around healthy lifestyle behaviors, which is vital given the prevalence of chronic disease.

It [the web-based module] raises for me the importance of the discussions around nutrition and physical activity and to not skim over them but spend some time doing that.

P09, PN

Other GPs perceived they already knew all the information presented in the web-based module and that it was not pitched appropriately for their profession. Two GPs felt that the web-based module would be more suitable for PNs and GP registrars due to the nature of the content.

All doctors more or less know all of that [information in the web-based module], so it would be interesting more for doctors in training, graduates, maybe.

P05, GP

It’s [the web-based module] pitched low for a GP really or pitched inappropriately for a GP. It’s not really understanding what happens in a consultation...perhaps for the practice nurses.

P10, GP

While thinking aloud, some participants said they experienced difficulties with wording questions related to lifestyle behaviors, highlighting the complexity of behavior change interventions. Participants perceived the simplified 5A’s model and examples of engaging in behavior change discussions would facilitate difficult conversations with their patients, particularly in initiating behavior change interventions.

I really like the questions to ask part here because I think one of the things I find challenging sometimes is how to word certain questions.... I actually noted a couple down so that way I can just remind myself to use them in my consults as a conversation starter.

P06, GP

It’s good that they give you a three-step model to help you apply the brief interventions instead of giving a whole bunch of information and no guidance for how to administer it.

P07, PN

Many participants expressed that the web-based module allowed them to upskill and reinforce their prior knowledge, which is essential to all health care professions.

The intensities of exercise and the subjective measures example.... I really like that because I get patients all the time who ask me OK, the guidelines say do moderately intense exercise.... I think we kind of always say, vigorous or moderately intensive and patients, the lay person doesn’t really know what that means...this sort of gives you a really good example of what that’s trying to say.

P06, GP

I think this is important and updating my clinical information, things like the waist measurement.... I thought it was 90 centimeters for women but it’s 80.

P09, PN

I suppose it’s [the web-based module] going to encourage me at actually to do waist circumferences a bit more.

P10, GP

#### Theme 3: Experiencing Barriers and Facilitators to Using the Healthy Lifestyles Suite

Participants appeared surprised and impressed that the Healthy Lifestyles suite was free of charge, given their recall that learning opportunities were usually expensive. The importance of free learning was highly emphasized by many participants and would directly alleviate the cost barrier.

Having a free service is pretty huge because most people have gone from uni [sic] to then having a job and they don’t really want to go to then spending more money out of their pocket on education. So I think free education is really important.

P01, PN

Participants voiced that the progress saving functionality offered them the opportunity to step away from the web-based module and return to it at their convenience, which appeared to alleviate time constraints. Progress saving reportedly provides flexibility, allowing for learning to be completed within shorter available time blocks during the working day.

The progress being saved, that’s good because I can jump out and come back to it [the web-based module], especially whilst I’m working...it’s good and not frustrating that you have to come back every time to the beginning. It’s nice that it’s saved.

P02, PN

GPs and PNs had opposing views regarding the time frame to complete the web-based module. Some participants believed they could complete the web-based module within the estimated time frame of 60 minutes. In contrast, others stated the web-based module would take longer than an hour but were comfortable spending more time on it due to its simplicity and richness of information. Participants expressed that lengthier web-based modules were overwhelming and brought up the perceived issue of time constraints associated with primary health care.

I like the module length for this one. It’s not too long, not too short, as in there are not too many components to complete to be able to finish it. I think sometimes when you’ve got more than 10 components for each part, you feel it can be a bit overwhelming.

P06, GP

I don’t know that I would complete it [the web-based module] in an hour only because there’s so much good information in it that I just want to take my time and read and digest and sort of consider how I might use that in a clinical setting.

P08, PN

## Discussion

### Principal Findings

This study explored GPs’ and PNs’ perceptions regarding web-based learning to support patients with behavior change. Qualitative inquiry explored the factors influencing GPs’ and PNs’ participation in web-based learning, including provider reputation, awareness of availability, resources, content quality, usability, cost, and time. Perceptions of quality web-based learning appears to be associated with culturally tailored information, resources, a balance of information and interactivity, plain language, user-friendly navigation, appealing visual presentation, communication examples, and simple models. Whereas free web-based learning that features progress saving and module lengths of less than 2 hours alleviate perceived cost and time barriers. These findings were generally consistent across the participants, except for some GPs’ comments.

Learning providers’ reputation and awareness of availability appear to impact GPs’ and PNs’ adoption of web-based learning. Several studies indicate GPs’ and PNs’ perceive that professional organizations have a lower reputation for learning [5,10]. Therefore, provider reputation may have a more significant impact on GPs’ and PNs’ perceptions than initially expected. Two studies found less than a third of GPs and PNs perceived awareness of learning opportunities as a barrier [2,5]. The Healthy Lifestyles suite is not currently advertised to GPs and PNs, which may explain the participants’ lack of awareness. GPs and PNs discover more learning opportunities through professional associations (58.6%) than health care organizations (33%) [5], which coincides with participants’ advertising suggestions. Advertising may play a crucial role in increasing GPs’ and PNs’ awareness of web-based learning opportunities. Queensland Health may benefit by advertising through the suggested professional organizations to communicate the availability of the Healthy Lifestyles suite to GPs and PNs.

Culturally tailored information and resources seemed to be appreciated by participants and increased perceptions of quality. Including Aboriginal and Torres Strait Islander information was described as beneficial to practice, highlighting the need to support learning for cultural competence in primary health care. Previous studies indicate that clinicians, including GPs and GP registrars value, but lack learning opportunities around Aboriginal and Torres Strait Islander populations [37,38], which is congruent with this study. However, these studies were not specific to web-based learning or behavior change. Many participants emphasized the resources in the web-based learning to facilitate behavior change interventions. Similarly, GPs’ work-related internet usage was higher for obtaining information for patients (93.5%), compared to learning (80.4%) [6]. This suggests information that is accurate, suitable, and culturally appropriate may be challenging to find, which was mentioned by a participant in this study. The Healthy Lifestyles suite contains Aboriginal and Torres Strait Islander information and resources, which may help to address the barrier of insufficient resources [5,15,18,20].

Perceptions of quality content in web-based learning appear to be associated with using plain language, a balance of information and interactivity and information accuracy. A study of GPs (n=12) evaluating a web-based module on vitamin D found that most participants strongly agreed that the plain language was easy to understand [39], which is reflective of this study. Many participants reported that the web-based module contained an appropriate balance of information and interactivity to facilitate learning. Several studies demonstrated greater satisfaction and comprehension from incorporating interactive elements in learning [11,40]. Another study indicated that GPs and PNs desire more interactivity and visual aids [5]. These studies did not examine web-based learning exclusively. The participants perceived the presented information as accurate, which appears to be related to statistics, references, prior knowledge, and experience. Australian GP registrars value referencing in web-based learning [41], which is reflective of this study and evidence-based practice. Furthermore, nurses use their knowledge and experience when evaluating information relevance and trustworthiness in web-based learning [42]. This is congruent with participant statements, suggesting that GPs and PNs critically evaluate information in learning. No data could be found for GPs’ and PNs’ perceptions of information accuracy relative to statistics. Therefore, plain language, the proportion of information and interactivity, and information accuracy may affect GPs’ and PNs’ perceptions of quality and engagement in web-based learning. The Healthy Lifestyles suite uses plain language, references, and reportedly has an acceptable balance of information and interactivity.

Visual presentation and usability were also reported indicators of quality web-based learning. Headers, fonts, spacing, colors, and pictures appeared to influence the perception of quality and facilitate engagement with the web-based module. Previous research indicates that visual presentation influences nurses’ perceived usefulness, ease of use, and enjoyment of web-based learning, which impacts intention to use [7]. These preferences are subjective and may be influenced on an individual level. Participants perceived the user-friendly navigation as a facilitator of web-based learning. A feasibility study of a web-based module with similar navigation to the Healthy Lifestyles suite was considered user-friendly by GPs [39]. A good structure and explicit instructions increase perceived functionality, facilitating more effective content navigation, producing a more enjoyable experience [7]. The Healthy Lifestyles suite appears to have an acceptable visual presentation and user-friendly navigation.

Many participants believed that the web-based module allowed them to upskill and reinforce their prior knowledge. A systematic review found that web-based learning modalities improved nurses’ knowledge, skills, attitudes, and self-efficacy, leading to improved performance [43]. These results are comparable with this study but are not based on web-based learning for behavior change. Many participants perceived that the web-based module’s communication examples and simple model facilitated their learning for behavior change interventions. Similarly, a behavior change learning program using the 5A’s model and motivational interviewing significantly increased GPs’ and PNs’ behavior change skill use [22]. Statistical analysis demonstrated improvements from baseline to clinical practice [22]. Qualitative exploration found a reduction in perceived barriers to behavior change interventions, including time constraints, patient resistance, and improved clinician-patient relationships [21]. Furthermore, web-based learning containing motivational interviewing examples improved nurses’ (n=31) perceived skill and self-reported skill use [4]. The Healthy Lifestyles contains communication examples and a simple model, which may help to facilitate professional knowledge and skill development for delivering behavior change interventions to patients.

Factors including cost and time were emphasized by participants as impacting learning. GPs and PNs want cost-effective learning options [20], but report many as expensive [9]. Similarly, participants in this study expressed cost as a significant barrier to learning. Perceived time constraints seemed to impact GPs’ and PNs’ ability to complete learning [8]. GPs and PNs want greater availability, backtracking, and to learn at their own pace [5], which is congruent with this study. The progress saving feature was reported to alleviate time constraints and facilitate web-based learning, particularly during work hours. Participants reported opposing views regarding their ability to complete the web-based module within the estimated time frame. A study of a vitamin D web-based module reported an average completion time of 124 minutes by GPs [39]. Feedback from participants (n=12) indicated that most (n=10) perceived the completion time as reasonable [39]. In contrast, web-based modules that required 6 hours to complete were too lengthy, even with progress saving [44]. The Healthy Lifestyles suite is free of charge, uses the progress saving feature and estimates an hour to complete, thereby appearing to be acceptable and feasible by participants.

### Recommendations for Future Research

Further research should be conducted to increase understanding of the effects of culturally tailored information, plain language, information accuracy, and communication examples for web-based learning. This study would benefit from further analysis to compare the cost and benefits to Queensland Health and the broader health system through GPs’ and PNs’ use of the Healthy Lifestyles suite, including changes to practice and patient health outcomes.

### Limitations

All interviews were conducted by a single researcher, which produced consistency in interviewing but increased the potential for researcher bias. The development of the interview protocol and 3 pilot interviews were done to reduce bias and reinforce the research methods. The overwhelming majority of the participants were female. While this was anticipated for PNs, it was not for GPs. Male GPs and PNs were underrepresented in the study, thereby possibly affecting the generalizability of the results. The lead researcher was unable to observe some participants using the web-based module due to an inability to share their screens. This impacted the researcher’s ability to view participants’ interactions with the web-based module and the timing of questions.

### Conclusions

This study highlights the factors and features influencing GPs’ and PNs’ adoption of web-based learning to support patients with behavior change. Influential factors include provider reputation, awareness of availability, resources, content quality, usability, cost, and time. GPs and PNs desire quality web-based learning that contains culturally tailored information, resources, a balance of interactivity and information, plain language, user-friendly navigation, appealing visual presentation, communication examples, and simple models. Free web-based learning that features progress saving and module lengths of less than 2 hours alleviate perceived cost and time barriers. The Healthy Lifestyles suite contains many of these features and appears highly acceptable and feasible by participants. While further research is required, learning providers may benefit by including these features in their future behavior change web-based learning for GPs and PNs.

## Appendix

Multimedia Appendix 1 Interview protocol.

## Abbreviations

GP: general practitioner
PHN: primary health network
PN: practice nurse
RACGP: Royal Australian College of General Practitioners

## Data Availability

The data sets generated during or analyzed during this study are available from the corresponding author on reasonable request.
