# Supplementary material for: Web-Based Learning for General Practitioners and Practice Nurses Regarding Behavior Change: Qualitative Descriptive Study
Source: JMIR Med Educ. 2023 Jul 27;9:e45587. doi: 10.2196/45587 (PMC10415945; doi:10.2196/45587)
Supplement: Multimedia Appendix 1 [file mededu_v9i1e45587_app1.pdf]

1. Were you aware that these learning modules were available prior to signing up for the study?
2. Is Queensland Health somewhere you'd usually look to for continual professional development opportunities?
3. Can you talk me through the process to create an account?
4. Can you describe for me what it was like navigating the module?
5. What do you think about having your module progress saved?
6. How would you describe the language used in the module?
7. Tell me about the interactive activities and videos? What effect do they have on your learning?
8. How accurate you do think the information being provided is?
9. What do you think about the amount of information that is being provided?
10. Overall, how would you describe the quality of the content in the module?
11. Is there anything else you think should be added to the module?
12. Can you describe the module length for me?
13. The module estimates an hour to be completed, how likely do you think it can be completed in that time frame?
14. This learning suite is free of charge, how does cost impact your choice for CPD?
15. How would you describe the value of this learning module?
16. How relevant is the topic of behaviour change to your practice?
17. What impact did this learning have on your practice?
